# Prevalence of Shiga Toxin-Producing *Escherichia coli* O157 in Wild Scottish Deer with High Human Pathogenic Potential

**DOI:** 10.3390/ani13172795

**Published:** 2023-09-02

**Authors:** Stephen F. Fitzgerald, Mairi C. Mitchell, Anne Holmes, Lesley Allison, Margo Chase-Topping, Nadejda Lupolova, Beth Wells, David L. Gally, Tom N. McNeilly

**Affiliations:** 1 Moredun Research Institute, Pentlands Science Park, Bush Loan, Edinburgh EH26 OPZ, UK; 2 Scottish *E. coli* O157/STEC Reference Laboratory, Department of Laboratory Medicine, Royal Infirmary of Edinburgh, 51 Little France Crescent, Edinburgh EH16 4SA, UKlesley.allison@nhslothian.scot.nhs.uk (L.A.); 3 The Roslin Institute and R(D)SVS, The University of Edinburgh, Easter Bush, Edinburgh EH25 9RG, UK

**Keywords:** Shiga toxin-producing *Escherichia coli* (STEC), O157, deer, prevalence, whole genome sequencing, public health

## Abstract

**Simple Summary:**

Shiga toxin-producing *E. coli* (STEC) serogroup O157 can cause serious infections in humans, with symptoms ranging from bloody diarrhoea to kidney failure, and, in some instances, can be life-threatening. The natural reservoir for these bacteria is livestock, particularly cattle; however, there is an increasing number of human cases associated with wildlife species such as deer. In Scotland, a human STEC O157 outbreak in 2015 that was associated with the consumption of venison prompted us to investigate the prevalence of STEC O157 in Scottish wild deer species. Although the estimated prevalence was low (0.28%), we found that STEC O157 isolates were shed at high levels from positive deer and that these isolates had the potential to cause severe disease in humans. Furthermore, retrospective analysis identified an isolate from this study as the likely source of another Scottish human outbreak in 2017.

**Abstract:**

Shiga toxin-producing *E. coli* (STEC) infections associated with wildlife are increasing globally, highlighting many ‘spillover’ species as important reservoirs for these zoonotic pathogens. A human outbreak of STEC serogroup O157 in 2015 in Scotland, associated with the consumption of venison meat products, highlighted several knowledge gaps, including the prevalence of STEC O157 in Scottish wild deer and the potential risk to humans from wild deer isolates. In this study, we undertook a nationwide survey of wild deer in Scotland and determined that the prevalence of STEC O157 in wild deer is low 0.28% (95% confidence interval = 0.06–0.80). Despite the low prevalence of STEC O157 in Scottish wild deer, identified isolates were present in deer faeces at high levels (>10^4^ colony forming units/g faeces) and had high human pathogenic potential based on whole genome sequencing and virulence gene profiling. A retrospective epidemiological investigation also identified one wild deer isolate from this study as a possible source of a Scottish human outbreak in 2017. These results emphasise the importance of food hygiene practices during the processing of wild deer carcasses for human consumption.

## 1. Introduction

Shiga-toxin producing *Escherichia coli* (STEC) are important zoonotic pathogens of public health concern worldwide [1]. STEC are characterised by their ability to produce Shiga-toxins (Stx), a primary virulence factor, which in humans can cause haemorrhagic colitis and, in severe cases, life-threatening haemolytic uremic syndrome (HUS) [2]. There are two major Stx toxin variants, Stx1 and Stx2, with additional Stx2 subtypes Stx2a–g [2]. STEC strains can encode either Stx1, Stx2 or combinations of both toxins or Stx2 subtypes. Stx2a is considered the most potent subtype and STEC strains encoding Stx2a are strongly associated with severe clinical outcomes, such as HUS, in the UK [3]. In Europe and North America, STEC serogroup O157 causes the most human cases [4]; however, other serogroups (O26, O45, O103, O111, O121 and O145) are also a threat to human health and there has been an increasing incidence of outbreaks associated with non-O157 STEC in recent years [5,6]. STEC O157 isolates are grouped into three genetically distinct lineages: Lineage I, Lineage I/II and Lineage II. In the UK, strains from Lineage I (Lineage Ic) of phage type (PT) 21/28 have been associated with severe human disease over the last decade [3], particularly in Scotland, which has a high incidence of STEC O157 infections (4–5 per 100,000), with almost 50% of these cases occurring in children under 16 years old and 85% of HUS cases occurring in this age group [7]. 

Cattle are considered the primary reservoir of STEC O157 and transmission to humans is incidental via direct bovine faecal contact or exposure to contaminated soil, vegetation, water or food products [8]. As such, prevalence and intervention studies have been biased towards understanding and controlling STEC within cattle populations. In Scotland, large-scale prevalence studies have demonstrated a consistent STEC O157 herd-level positivity of ~20% and individual pat prevalence on positive farms of 5–10% over the past thirty years [9,10]. Furthermore, it was estimated that <20% of animals on positive farms are ‘super-shedders’ (>10^4^ colony forming units per gram of faeces (CFU/g)); however, these few animals account for the majority of STEC O157 shedding on farms and ~80% of all animal-to-animal transmissions [11]. 

Other ruminants, such as sheep, goats and deer, can also act as reservoirs for STEC [12]; however, there is limited information on the prevalence of STEC in other ruminant species, and what risk they pose to human health. In 2015, a clinical outbreak of STEC O157 occurred in Scotland that was associated with the consumption of venison products [13]. Epidemiological investigation of the twelve human cases from this outbreak, five of which required hospitalisation but fully recovered, indicated that the consumption of venison products (steaks, grillsteaks, sausages and meatballs) supplied to several retailers by a single approved game handling establishment (AGHE) was the likely source of infection. However, the clinical outbreak strain, a phage type 32 (PT32), could not be isolated from these products. Investigation of this outbreak highlighted knowledge gaps relating to the risk of STEC O157 infection associated with venison consumption, including (i) a lack of STEC O157 and other STEC serotype prevalence data in Scottish deer populations; (ii) whether deer, like cattle, can act as STEC O157 super-shedders; and (iii) the virulence potential of STEC O157 deer isolates. 

The aim of this study was to provide an accurate estimate of STEC O157 prevalence in Scottish wild deer destined for the human food chain, including levels of STEC O157 in deer faeces, and determine their pathogenic potential and genetic relatedness to STEC O157 strains found in UK human infections and British cattle.

## 2. Materials and Methods

### 2.1. Sampling Methodology

A sample size calculation was carried out based on 10 years of historical data obtained from Forestry and Land Scotland (FLS) (see Supplementary Material S1 and as described in [14]). The data represented the annual number of deer culled in Scotland, categorised by species, through FLS larders. Over the course of ten years, the average number of culled deer was 24,674 (95% CI: minimum 19,059; maximum 29,511). To ensure a 95% probability of accurately estimating the true prevalence of STEC O157 in Scottish wild deer, a sample size of 1521 was determined to be required. Given the consistency of the data across the 10-year time frame, the sampling design described below was based on the 2016/2017 data. Table S1 illustrates the distribution of culled deer by month and species during this period, showing peak culling in October/November, which remained relatively high throughout the winter. Roe deer accounted for approximately 52% of the culled deer, followed by red deer at 38%, sika deer at 9% and less than 2% from fallow deer.

In total, 1888 sample packs containing sterile gloves and sample pots were provided to stalkers recruited by Deer Management Groups (DMGs) and FLS. Stalkers collected faecal samples directly from the rectums of culled deer using sterile gloves and stored them in sterile plastic 50 mL pots. All samples were placed in Category B Biological Samples UN3373 sample boxes and transported to the Moredun Research Institute (MRI) for analysis. To ensure that a representative sample was obtained from the wild deer population of Scotland, factors such as the time of year, sample location, deer species and sampling personnel (DMGs or FLS) were integrated into the sampling plan (see Supplementary Material S2). Additionally, the deer stalkers completed a questionnaire at the time of sampling, providing details on the location (UK National Grid Map Reference), number of the cull site, species, estimated age of the culled deer and evidence of co-grazing with other herbivores based on the location of the cull site (Supplementary Material S3). Grid reference data were used to map sampling sites using the QGIS 3.10 software [15].

### 2.2. Isolation of E. coli O157

Captivate™ O157 immuno-magnetic beads were used to isolate *E. coli* O157 from 1 g of deer faecal separation, as previously described [14,16]. Following immuno-magnetic separation, *E. coli* O157-positive colonies were confirmed by latex agglutination using an *E. coli* O157 Latex Test Kit (Oxoid). To enumerate *E. coli* O157 colonies, 10-fold serial dilutions of positive faecal samples were made in PBS, dilutions were plated in duplicate on CT-SMAC agar plates and positive colonies were counted after 24 h incubation of plates at 37 °C. Counts were expressed as colony forming units per gram of faeces (CFU/g) as previously described [8], and the limit of detection was 50 CFU/g. 

### 2.3. Sequence Analysis of E. coli O157 Isolates

*E. coli* O157 isolates identified in deer faeces were subject to phage typing and whole genome sequencing (WGS) at the Scottish *E. coli* O157 STEC Reference Laboratory (SERL) [17]. For WGS, DNA was extracted using the DNeasy Blood and Tissue kit (Qiagen, Crawley, UK) following a prelysis step, as recommended by the manufacturer. Libraries were prepared using the Nextera XT DNA kit (Illumina, Cambridge, UK) and paired-end sequencing was performed on the Illumina MiSeq using 500 cycle v2 reagent kits to produce 2 × 250 bp reads. All sequencing reads were uploaded to the NCBI Sequence Read Archive (SRA) (PRJNA419720). Fastq files were analysed using the Scottish Microbiology Reference Laboratories WGS Pipeline (SMiRLWBP) and BioNumerics v 7.6 (Applied Maths), using the wgMLST and the *E. coli* genotyping plug-in tools, as previously described, with some modifications [17]. In brief, the SMiRLWBP used KmerID to identify bacterial species [18] and GeneFinder (M. Doumith, unpublished data) to map reads to a panel of serotype and virulence genes using Bowtie 2 [19]; in silico predictions that matched to a gene determinant at >80% nucleotide identity and >80% target gene length were accepted. MLST alleles (*adk*, *fumC*, *gyrB*, *icd*, *mdh*, *purA*, *recA*) and sequence types (STs) were determined using Metric-Oriented Sequence Typer (MOST; [20]) and Shiga toxin gene subtyping was performed using a combined mapping and BLAST approach, as previously described [21]. STEC O157 were reference mapped to *E. coli* O157 strain Sakai GenBank accession no. BA000007.2 using BWA and GATK2 to identify variants, as previously described [3]. Positions the same as the reference were removed and a JSON file with variant positions and ignored positions was sent to UKHSA to obtain SNP addresses using SnapperDB [22] (Table S3).

In BioNumerics v 7.6, fastq files were processed using the calculation engine and the wgMLST client plug-in. Assembly-free and assembly-based allele detection for cgMLST (2513 core loci synchronised with EnteroBase schema) analyses were performed. The assembly was performed using SPAdes and basic assembly metrics were calculated (Table S3). The average read coverage for the deer samples ranged from 63× to 133× and the *N*_50_ > 300 scores ranged from 146,894 bp to 193,455 bp. The assembled genomes were analysed using the *E. coli* genotyping plug-in, which contains databases for serotype, virulence and resistance prediction obtained from the Center for Genomic Epidemiology (www.genomicepidemiology.org/services). The detection parameters for gene detection using BLAST were set to 90% sequence identity and 60% sequence coverage. 

A cgMLST analysis was performed to identify STEC O157 from human clinical infections that most closely matched the STEC O157 isolated from deer. A more detailed phylogenetic analysis of core genome sequence data was also performed, as described previously [23], to determine the relationship between deer STEC O157 isolates and those identified in British cattle destined for the human food chain and a larger dataset representative of UK human clinical cases [3,9,10]. Single nucleotide polymorphisms (SNPs) within the core sequences were identified and aligned to the reference genome *E. coli* O157 strain Sakai (ref number: GCF_000008865.2). The phylogenetic tree was constructed using the FastTree software v 2.1.10 [24] and visualised with ITOL [25]. 

### 2.4. Prevalence Estimates of STEC O157 in Scottish Wild Deer

As a result of the low number of deer positive for STEC O157, prevalence was presented using a binomial proportion in StatXact version 11 (Cytel Software Corp., Cambridge, MA, USA). The exact confidence interval was constructed using the Clopper–Pearson method [10]. 

## 3. Results

### 3.1. Deer Sampling

**Figure 1:**
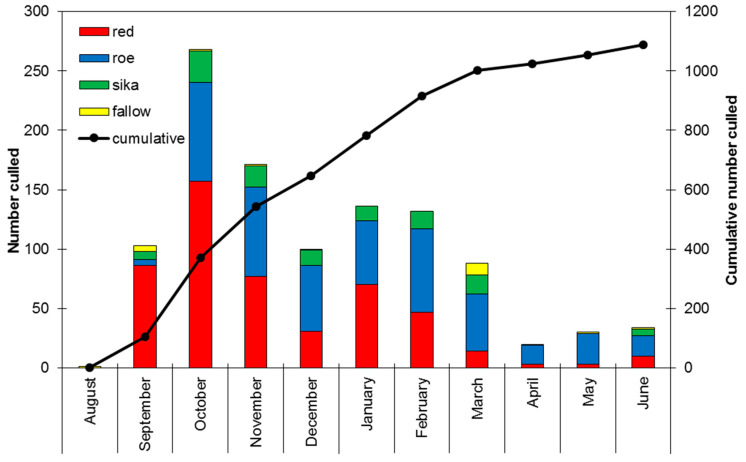
Numbers of Scottish deer sampled per month during the study. The total (primary *y*-axis) and cumulative (secondary *y*-axis) number of deer culled per month is shown. The proportion of red deer (red), roe deer (blue), sika deer (green) and fallow deer (yellow) culled is shown for each month. The cumulative number of deer culled and sampled is indicated by the black line.

A sampling plan was designed to be representative of a full deer culling year in Scotland, with the first and last samples received on 31 August 2017 and 21 June 2018, respectively. In total, 1888 sample packs were distributed to DMGs and FLS across Scotland, from which a total of 1087 samples were returned for analysis, representing a return rate of 58%. In agreement with previous years, peak culling occurred in October–November and this was reflected in the number of samples received; see Figure 1.

**Figure 2:**
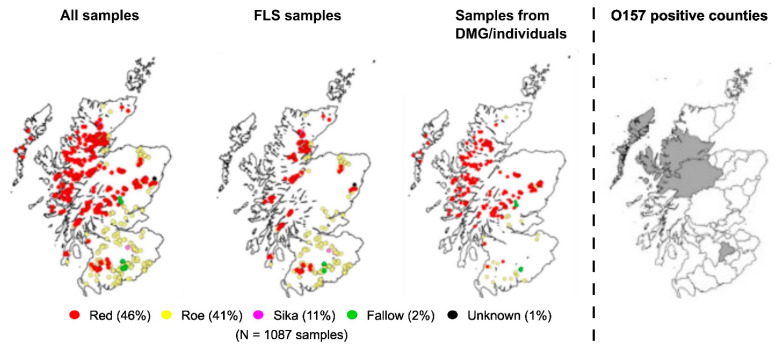
Geolocation of deer cull sites and STEC O157-positive deer. Geolocations and species (coloured dots) for total deer culled, deer culled by Forestry and Land Scotland (FLS) and deer culled by Deer Management Groups (DMG) or individuals are shown. Counties within Scotland where a single STEC O157-positive deer was identified are highlighted in grey.

Analysis of samples by cull site showed an even distribution of deer sampling throughout Scotland (Figure 2), with the majority of red deer sampled in North and North-West Scotland and roe deer in South and South-East Scotland. The distribution of deer species sampled for this study was 41.5% roe deer, 46.0% red deer, 10.6% sika deer and 1.9% from fallow deer. This was comparable to that culled in 2016/2017 by FLS, although proportionally more red deer were sampled (46.0% versus 37.6%, Table S2). 

### 3.2. Prevalence of STEC O157 Strains Isolated from Scottish Wild Deer

Of the 1087 deer samples received, eight (0.74% (95% CI = 0.32–1.45) were positive for *E. coli* O157. However, only three (0.28% (95% CI = 0.06–0.80) of these isolates were *stx*-positive (as determined by WGS). The cull location (by county) of the STEC O157-positive deer is shown in Figure 2 and details of the three STEC O157 *stx*-positive isolates are summarised in Table 1. Two STEC O157-positive red deer were culled in Inverness-shire and Ross and Cromarty, respectively, and a third positive Sika deer was culled in Peebles-shire. A history of co-grazing land with livestock (sheep/cattle) was only reported for a single O157 *stx*-positive red deer culled in the Highlands. Enumeration of STEC O157 from faecal samples of all three positive deer demonstrated that each animal was shedding levels of STEC O157 >10^4^ CFU/g faeces at the time of sampling, indicative of these animals being ‘super-shedders’.

### 3.3. Virulence Potential of STEC O157 Strains Isolated from Scottish Wild Deer

To further characterise the Scottish *E. coli* deer isolates, all eight STEC O157 were phage-typed and whole-genome-sequenced. The genomes of three *stx*-positive isolates were also compared with those held in the SERL sequence database (n = 2662), which included the 2015 Scottish venison outbreak strain (SME-19-101).

Phage typing identified both red deer STEC O157 isolates (SME170024 and SME170026) as PT21/28 and the single Sika deer STEC isolate (SME170025) was typed as PT8; the five *stx*-negative *E. coli* O157 isolates reacted with a phage but did not conform to a recognised pattern (RDNC). All three STEC O157 *stx*-positive deer isolates were genotyped as H7, ST11, were positive for the intimin gene, *eae*, and carried the gene for the *stx2a* toxin subtype (Table 1). One red deer STEC O157 isolate (SME170024) also carried the gene for the *stx2c* toxin subtype. Of the five *stx*-negative isolates, one was O157:H7 ST11, one was O157:H39 ST1041, two were O157:H43 ST155 and one was O157:H42 with a novel sequence type ST9986. Only the O157:H7 and O157:H39 *stx*-negative isolates were positive for *eae* (Table S3).

**Figure 3:**
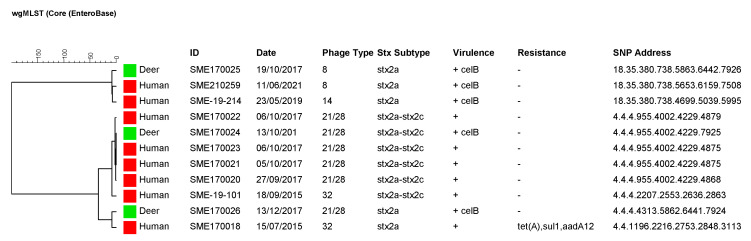
Dendrogram based on the allelic profiles of 2513 cgMLST genes for the three deer STEC O157 (green) and eight related human STEC O157 strains (red). The tree was created in BioNumerics v7.6 with the categorical (differences) coefficient of similarity (no scaling) and complete linkage clustering. The columns from left to right are the Strain ID, date received by SERL (human) or cull date (deer), phage type, *stx* subtype, virulence genes detected, where + denotes the presence of the genes *astA*, *eae*, *espA*, *espB*, *tir*, *ehxA*, *katP*, *espP*, *etpD*, *espF*, *FimH*, *hlyD*, *iha*, *iss*, *nleA*, *nleB*, *nleC*, *toxB*, *espJ*, *gad*, *tccP*, antimicrobial resistance gene profile and SNP address.

To determine the genetic relatedness of the deer STEC O157 isolates identified in this study with isolates from Scottish human cases of infection, a cgMLST analysis was performed and SNP addresses were determined for those that most closely clustered together. Most significantly, the results showed that the PT21/28 *stx2a–stx2c*-positive deer strain (SME170024) was ≤1 cgMLST alleles and fell within the same five-SNP cluster as four human PT21/28 *stx2a–stx2c*-positive isolates involved in an outbreak in October 2017 (Figure 3). A five-SNP threshold was used to putatively link STEC cases to the same temporally related cluster. Epidemiological investigations at the time did not identify a putative source of infection; however, a retrospective analysis showed that the time frame and geographical location of the deer sampling coincided with the travel history of the cases to the area, indicating the deer as a potential source of infection (Public Health Scotland, personal communication). The 2015 outbreak strain (SME-19-101) fell within the same 50-SNP cluster as the deer strain SME170024 but was PT32 *stx2a*-positive and differed at seven cgMLST loci. The other PT21/28 *stx2a*-positive deer (SME170026) clustered most closely with a PT32 *stx2a*-positive human strain (SME170018) differing at eight cgMLST and within a 100-SNP cluster threshold. The PT8 *stx2a*-positive deer strain (SME170025) fell within the same 25-SNP cluster as two human strains, a PT8 *stx2a*-positive (SME210259) and a PT14 *stx2a*-positive (SME-19-214) strain that differed by seven and eight cgMLST loci, respectively. None of the STEC O157 deer strains carried AMR genes and the only difference in virulence gene content (excluding the *stx* subtype) was the presence of *celB*, which interestingly was detected in all deer strains but only two human strains. However, as this is a plasmid-encoded gene [26], it is possible that this was related to the plasmid extraction efficacy during the preparation of DNA for WGS.

A whole genome phylogenetic analysis was also undertaken using available WGS data for UK human and bovine STEC O157 isolates to determine the likely origin of the Scottish wild deer isolates (Figure S1). Rather than forming a deer-specific cluster, all deer isolates clustered with bovine and human isolates in defined lineages, suggesting a bovine or non-deer origin for each of the deer isolates.

## 4. Discussion

The 2015 STEC O157 outbreak associated with the consumption of wild venison in Scotland highlighted the need to understand the prevalence of this zoonotic pathogen in Scottish wild deer. In an unpublished study conducted in 2003 (Singh BK, University of Edinburgh), no STEC O157 was detected in a total of 784 faecal samples collected from mainly wild Scottish deer of a range of species, including roe, red and sika deer. 

Our study showed that the prevalence of STEC O157 in Scottish wild deer destined for the human food chain remains low, with only three out of 1087 faecal samples (0.28%) being positive for the bacteria. This is substantially lower than the prevalence in Scottish cattle entering the food chain, which was recently estimated in 2014/15 to be 23.6% (16.6–32.5%) for a herd in Scotland and 10.6% (6.7–16.3%) for pats within a herd [10]. The low prevalence of STEC O157 in wild Scottish deer is in line with similar studies conducted in North America, Europe and Japan, which estimated the prevalence of STEC O157 in deer based on *stx* PCR positivity between 0 and 3% [27,28,29,30]. This finding supports the hypothesis that, unlike in cattle, STEC O157 may be unable to persist in wild deer populations and detection in this reservoir represents ‘spill-over’ from cattle and/or other livestock. 

In support of this, our phylogenetic analysis showed that Scottish wild deer *E. coli* O157 isolates did not form a deer-specific cluster. Instead, isolates clustered within clades containing UK bovine and human isolates, suggesting a bovine origin, or at least a non-deer origin, for these isolates, and transmission to deer is likely the result of co-grazing habitats with cattle. Similar clustering has also been observed for non-O157 STEC isolated from cattle and deer sharing the same agroecosystem in Michigan, USA [31]. Furthermore, 2/3 deer O157 isolates from our study were typed as phage type PT21/28, a dominant PT present in Scottish cattle for over 20 years [10], and both were closely related to cattle strains. Proving such transmission is difficult due to the low prevalence of STEC O157 in deer and would require further WGS data of STEC O157 deer strains for comparison with STEC O157 strains in the Scottish cattle population, ideally with strains isolated from individuals known to share the same environment. However, Frank et al. (2019) [32] and Singh et al. (2015) [31] have previously demonstrated the transmission of non-O157 STEC between cattle and wild deer sharing the same agroecosystems, suggesting that the similar transmission of STEC O157 is also likely to occur. 

Despite the low prevalence of STEC O157 in wild deer worldwide, significant outbreaks associated with the handling and/or consumption of contaminated wild deer products or produce contaminated with deer faeces have occurred in America [33], Japan [34] and Scotland [13]. Where investigated, isolates from each outbreak were found to encode the *stx2* Shiga toxin variant [13,33,34]. The severity of disease in humans is strongly correlated with the Stx subtype carriage and strains expressing the *stx2a* subtype are more likely to result in systemic sequelae [3,35]. In an analysis of UK clinical cases, the *stx2a* subtype was also shown to be a pre-requisite for the development of HUS, and *in vitro* assays have shown that Stx2a is 1000 times more toxic than the Stx1 variant to human renal cells [3,36]. The three deer STEC O157 isolates identified in this study were all positive for both *stx2a* and *eae*, a gene profile that is associated with high pathogenic potential and more severe forms of human disease, such as bloody diarrhoea and HUS [35]. The strains were also of two phage types, PT21/28 and PT8, which represent the two most frequently reported phage types associated with human STEC O157 infections in Scotland [6]. Of the two phage types, PT21/28, which was isolated from two red deer, was previously shown to cause more severe human disease in Scottish children [37]. Therefore, although the prevalence of STEC O157 in Scottish wild deer is low, the pathogenic potential of the strains that they carry is high and they pose a threat to public health. Indeed, our cgMLST and SNP clustering analyses demonstrated that all STEC O157 strains in this study differed by only eight or less cgMLST loci (8/2513) to known human isolates and fell within SNP clusters of 100 or less. Significantly, our sequencing analysis showed that strain SME170024 was highly similar to a strain that caused a Scottish STEC O157 outbreak in 2017, where three out of four cases went wild camping in the area and drank water from streams but did not eat deer products (Public Health Scotland, personal communication). This suggests that environmental contamination by deer faeces was a potential source of the infection. As comprehensive environmental sampling was not undertaken in this outbreak investigation, it is possible that both deer and human infections resulted from a common source of infection, such as grazing cattle. The high levels shed by the deer and the fact that no cattle grazing was noted in the area where the deer was culled, however, suggest that the deer was a likely source of the STEC O157 infection.

Interestingly, all STEC O157-positive deer in this study were found to be ‘super-shedding’ at the time of isolation, with levels of bacteria >10^4^ CFU/g faeces. ‘Super-shedding’ in Scottish cattle is strongly correlated with the carriage of both PT21/28 strains and strains that carry the Stx2a toxin variant [38]. All three Scottish deer isolates were *stx2a*-positive and 2/3 were typed as PT21/28. The estimated ‘super-shedder’ prevalence within STEC O157-positive cattle herds is between 9 and 20% [11,39,40]. Despite this low prevalence, however, ‘super-shedders’ can contribute > 90% of the total STEC O157 burden in feedlot pens and have been predicted to account for 80% of animal-to-animal transmission [39,40,41]. Wild deer have also been shown to shed STEC for prolonged periods of time, with estimated shedding times of 26 and 42 days for experimentally infected deer and wild deer, respectively [31,32]. Such prolonged shedding, coupled with our finding that wild deer can also become ‘super-shedders’, suggests that, when infected, wild deer can contribute significantly to the environmental prevalence of STEC O157 and are an important species in the circle of transmission between domestic livestock, wildlife and humans. However, given the low prevalence of STEC O157 in wild deer, it suggests that while super-shedding deer can contribute to environmental bacterial contamination, there does not appear to be strong evidence of significant deer-to-deer transmission. Deer ‘super-shedding’ would also directly increase the risk of STEC infection to humans due to the increased risk of contaminating the carcases of both the ‘super-shedder’ itself and cross-contamination of other carcases during the processing of venison products for human consumption. Wild deer are shot and the abdominal and pelvic viscera removed (i.e., gralloched) on the hillside, and there is theoretically a greater risk of faecal contamination of wild deer carcasses compared to farmed deer carcasses, which are usually processed at abattoirs. Indeed, a previous UK-based study demonstrated that retail packs of wild venison were more likely to be contaminated with *E. coli* than venison from farmed animals [42]. Therefore, strict hygiene precautions should be taken to avoid contamination of the carcass with faeces during gralloching and the downstream processing of the carcass. A recent microbiological study of ~100 Scottish wild deer carcasses found that pan-*E. coli* counts were within acceptable limits in around 50% of the carcasses sampled [43]. This indicates that the current guidelines for processing wild deer carcasses can be effective at limiting *E. coli* contamination, but that further improvements in carcass processing—for example, training in good hygiene practices—are required. 

Although domestic ruminant livestock are recognised as the primary reservoirs of STEC, particularly O157, human STEC infections arising from wildlife are increasing globally [44,45]. A recent review found that studies investigating wild deer as a reservoir accounted for 41.7% of all studies, highlighting wild deer as an important reservoir for STEC [45]. Further prevalence studies in the UK and other countries targeting wild deer and other potential wildlife reservoirs of STEC are required to understand the transmission dynamics between domestic livestock, wildlife and humans with a view to developing effective risk mitigation strategies. 

## 5. Conclusions

Our survey found that the prevalence of STEC O157 in Scottish wild deer populations was low (0.28%), in agreement with similar studies worldwide. Genotypic characterisation of the STEC O157 strains isolated from deer, however, demonstrated that these strains were of high pathogenic potential to humans, and the retrospective investigation of a Scottish outbreak in 2017 identified one deer isolate as a potential source of infection. Although cattle were identified as the likely source of Scottish wild deer isolates, our finding that infected deer can become ‘super-shedders’ indicates that wild deer can contribute significantly to environmental levels of STEC and are therefore an important species in the circular transmission between domestic livestock, other wild animals and humans.

## Figures and Tables

**Table 1:** Details of STEC O157 strains isolated from wild Scottish deer in 2017/2018.

Strain ID	County	Species	Sex	Age (Years) *	Co-Grazing History	PT	H-Type	*stx* Subtype	*eae*	Count (CFU/g Faeces)
SME170024	Inverness-shire	Red	F	4	None reported	21/28	7	*stx*2a and *stx2c*	+	1.0 × 10^4^
SME170025	Peebles-shire	Sika	F	5	None reported	8	7	*stx*2a	+	5.0 × 10^6^
SME170026	Ross and Cromarty	Red	M	1.5	Cattle/sheep	21/28	7	*stx*2a	+	7.7 × 10^7^

ID = identity; * age estimated by deer stalker; PT = phage type.

## Data Availability

Whole genome sequence data are in submission to NCBI. Other data presented in the study are available in the article and Supplementary Materials.

## Supplementary Materials

The following supporting information can be downloaded at: https://www.mdpi.com/article/10.3390/ani13172795/s1: Table S1: Number of deer culled by FLS in 2016/2017 by month and species; Table S2: Comparison of deer species sampled in this study with proportion culled by FLS in 2016/2017; Table S3: WGS data; Supplementary Material S1: Sample size calculation; Supplementary Material S2: Deer sampling strategy; Supplementary Material S3: Questionnaire completed by deer stalkers at time of sample collection; Figure S1: Phylogenetic analysis of STEC O157 deer strains with UK human and bovine cattle STEC O157 strains using core genome sequence data.
