# Supplementary material for: Prevalence of Shiga Toxin-Producing Escherichia coli O157 in Wild Scottish Deer with High Human Pathogenic Potential
Source: Animals (Basel). 2023 Sep 2;13(17):2795. doi: 10.3390/ani13172795 (PMC10486872; doi:10.3390/ani13172795)
Supplement: Supplementary file 1 [file animals-13-02795-s001.zip › Supplementary.pdf]

Supplementary Table S1 Number of deer culled by FLS in 2016/2017 by month and species.

Month	Overall	Fallow Deer	Red Deer	Red/Sika Deer (Hybrid)*	Roe Deer	Sika Deer
April	567	1	194	0	333	39
May	1713	10	559	1	1051	92
June	1217	9	358	2	761	87
July	832	3	322	0	437	70
August	291	1	96	0	179	15
September	793	3	384	0	142	264
October	5403	72	2416	3	2432	480
November	4635	98	1883	1	2259	394
December	2965	57	925	1	1729	253
January	3476	64	1288	1	1873	250
February	3324	66	1248	0	1734	276
March	3634	77	1164	0	2043	350

*reported by the stalker based on deer appearance.

Supplementary Table S2 Comparison of deer species sampled in this study with proportion culled by FLS in 2016/2017

Species	Deer species culled by FLS# (2016/2017)		Deer species culled and sampled in this study (2017/2018)	
	**N***	**%**	**N***	**%**
Red	10,837	37.6	498	46.0
Roe	14,973	51.9	449	41.5
Fallow	461	1.6	115	1.9
Sika	2,570	8.9	21	10.6

*hybrid deer were reported in the historical data (n=9) but are not included in the table above.

#FLS = Forestry and Land Scotland

Supplementary Material S1: Sample size calculation

Sample size for the study was estimated using the following formula:

$$n = (z^2 \times p(1-p))/d^2$$

where:

n= sample size

z=1.96 (z statistic for 95% level of Confidence)

p=prevalence estimate (here *p*=0.01)

d= precision, estimated as *p*/2 (here *d*=0.005)

Supplementary Material S2: Deer sampling strategy

In total, 800 sample packs were distributed to Scottish deer management groups (DMGs) and 1088 sample packs were distributed to 18 Forestry and Land Scotland (FLS) larders. The distribution of DMGs (red dots) and FLS larders (pie charts) are indicated in the figure below. FLS larders chosen for this study are designated with a white asterisk. Sampling from FLS larders was weighted for species, sex, month, and larder size. Sampling targeted three large larders (>600 carcasses/year) and three small larders (<600 carcasses/year) from the north and central regions of Scotland, and four large larders and two small larders for the Southern region of Scotland.

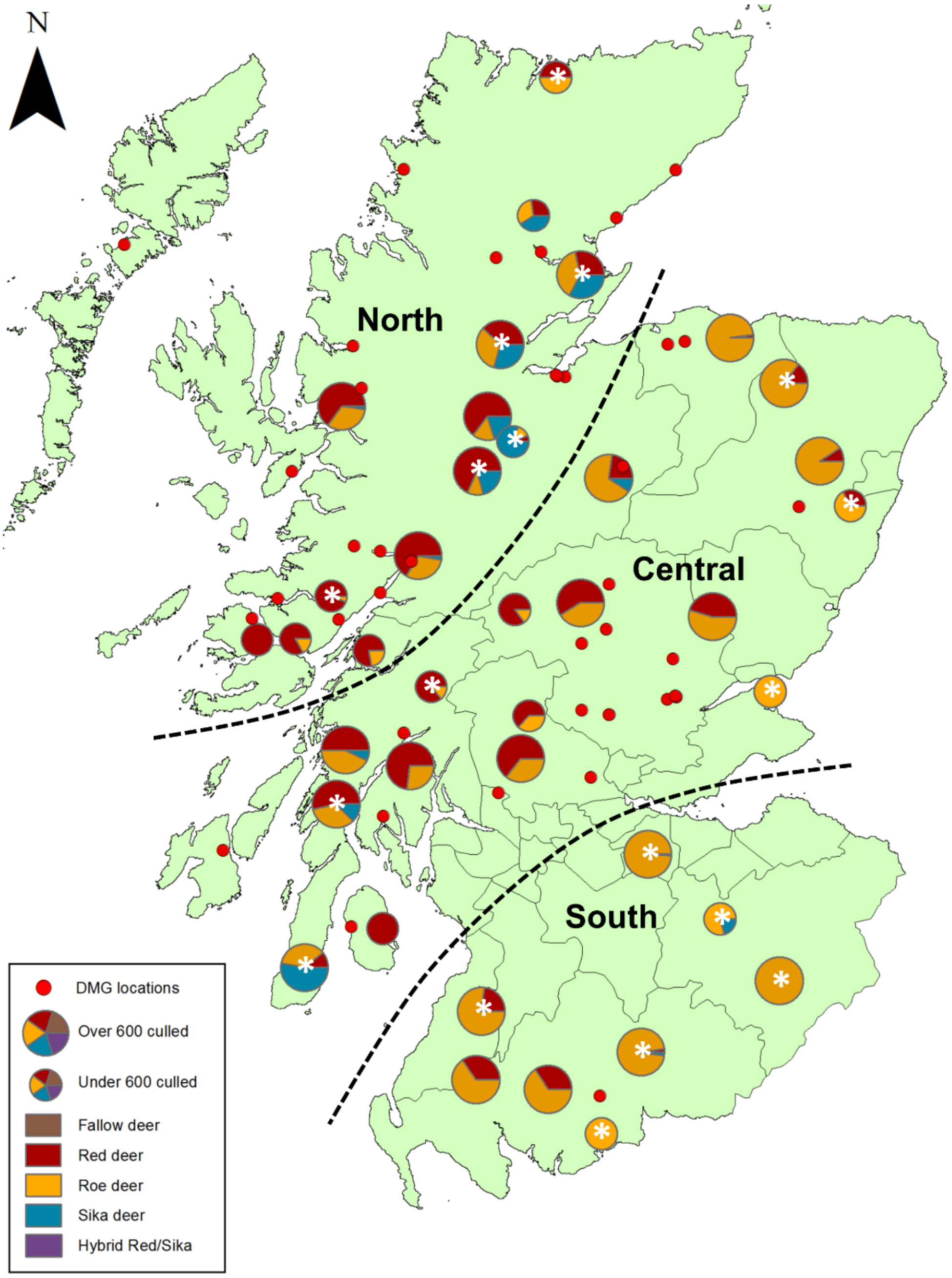
N
North
Central
South
DMG locations
Over 600 culled
Under 600 culled
Fallow deer
Red deer
Roe deer
Sika deer
Hybrid Red/Sika

Supplementary Material S3: Questionnaire completed by deer stalkers at time of sample collection.

Scottish Deer Health Survey 2017-2019

Moredun

QUESTIONNAIRE

**Date**	
**Time**	
**Tag number (if available)**	
**OS Reference of cull site**	OS Sheet:____________________________<br>6-digit grid reference:________________________
**Larder Address**	
**Deer species**	Red ☐ Roe ☐ Sika ☐ Other ☐<br>If other provide details:____________________________
**Gender**	Male ☐ Female ☐
**Condition Score**	1 ☐ 2 ☐ 3 ☐ 4 ☐ 5 ☐<br>1 = very poor condition; 5 = 'very good condition
**Estimated age**	___________years
**Shared range with other livestock/ wild herbivores**	Cattle ☐ Sheep ☐ Wild herbivores ☐<br>If wild herbivores provide details below:<br>________________________________
**Other comments**	

THANK-YOU FOR PARTICIPATING IN THIS SURVEY

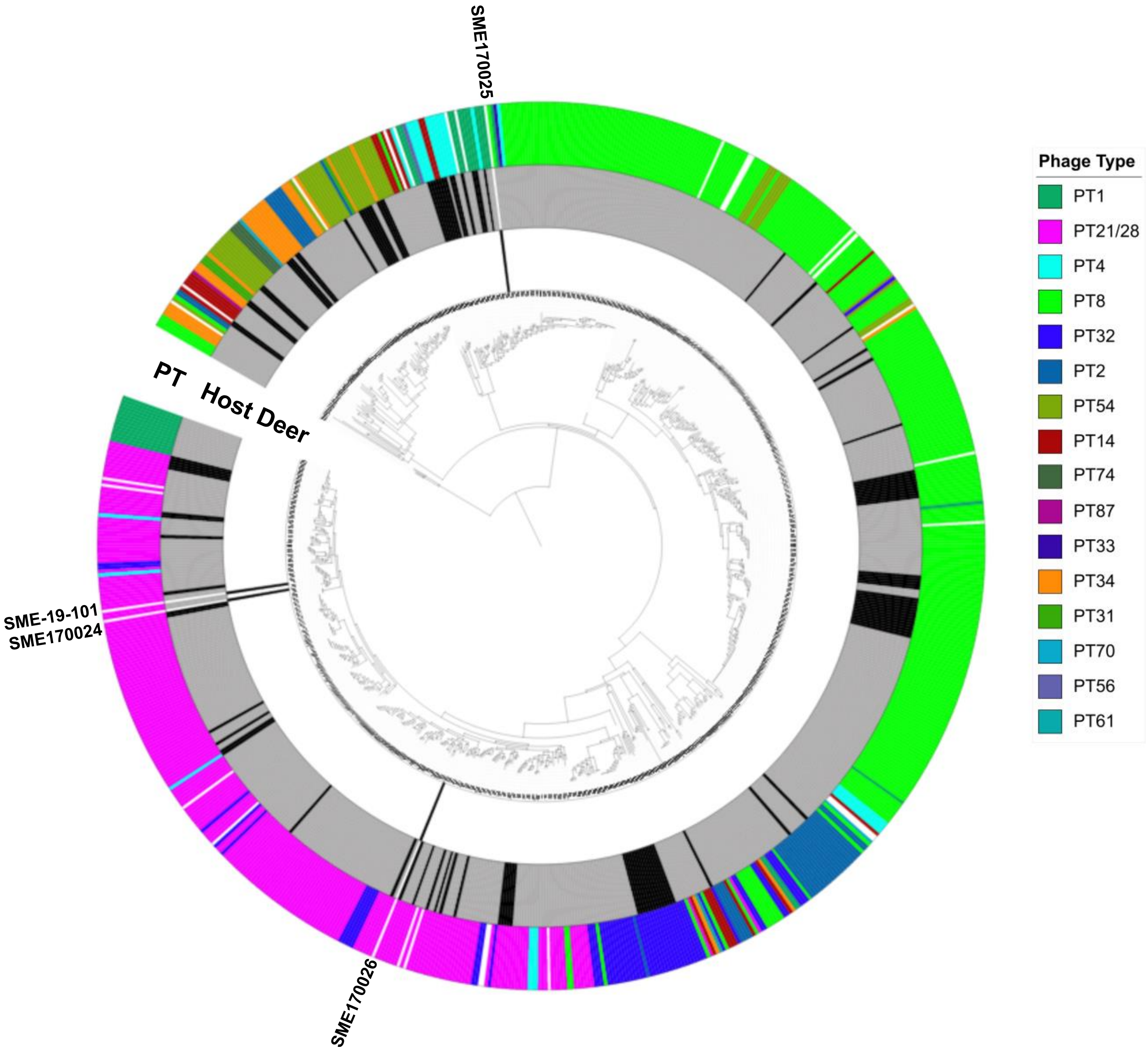

Supplementary Figure S1. Core genome phylogenetic analysis of STEC O157 strains from Scottish wild deer. Whole genome sequences of UK human, bovine and Scottish wild deer STEC O157 strains were used to generate the phylogeny**.** The phage type (PT) of each strain is shown in the outer ring. White bands indicate deer STEC O157 strains and strains that reacted with phage but did not conform (RDNC), were untypable or for which no PT information was available. Host species from which strains were isolated are shown in the middle ring (Cattle (black), Human (grey)) and inner rings deer (black). Strain identifiers for Scottish wild deer strains from this study and the 2015 venison outbreak are indicated.
